# Integrated Liver Transcriptomic and Proteomic Analysis Reveals Resistance Mechanisms Against *Pseudomonas plecoglossicida* in *Larimichthys crocea*

**DOI:** 10.3390/ijms27167208

**Published:** 2026-08-12

**Authors:** Ting Ye, Jiajie Zhu, Xiao Liang, Dandan Guo, Yilian Zhou, Bao Lou, Feng Liu

**Affiliations:** 1 State Key Laboratory for Managing Biotic and Chemical Threats to the Quality and Safety of Agro-Products, Institute of Hydrobiology, Zhejiang Academy of Agricultural Sciences, Hangzhou 310021, China; 15tye@stu.edu.cn (T.Y.); zhujiajie1225@163.com (J.Z.); liangx@zaas.ac.cn (X.L.); guodd@zaas.ac.cn (D.G.); zhouyl@zaas.ac.cn (Y.Z.); 2 Zhejiang Key Laboratory of Coastal Biological Germplasm Resources Conservation and Utilization, Wenzhou 325005, China

**Keywords:** *Larimichthys crocea*, disease-resistance, multi-omics, liver, *Pseudomonas plecoglossicida*

## Abstract

Visceral white-nodules disease (VWND), caused by *Pseudomonas plecoglossicida*, poses a severe threat to the large yellow croaker (*Larimichthys crocea*) aquaculture industry. Although breeding resistant strains is a promising strategy, the molecular basis of disease resistance in this host remains poorly understood. Here, 1500 fish were artificially infected, and extreme phenotypes (30 resistant, RL; 30 susceptible, SL) were selected based on survival time and liver pathogen load. Liver histopathology revealed that RL fish maintained intact architecture with only mild vacuolation, whereas SL fish exhibited widespread necrosis, inflammation, and hemosiderin deposition. Consistently, RL fish showed lower MDA levels and higher GSH-Px activity and TAC. Transcriptomic analysis identified 172 differentially expressed genes (DEGs): RL fish were characterized by upregulation of anti-inflammatory and tissue-protective genes (*Epo*, *CAV3*) and downregulation of pro-coagulant factors (*PAI1*, *K1kb1*). Proteomic analysis identified 111 differentially expressed proteins, with significantly enriched pathways including the peroxisome, pentose phosphate, and phagosome pathways. Integrated cross-omics analysis revealed eight co-enriched KEGG pathways; among them, arginine/proline metabolism, phagosome, oxidative phosphorylation, and focal adhesion were consistently upregulated in the RL group. These findings suggest that effective resistance to VWND in *L. crocea* may involve a coordinated, multi-layered defense program encompassing redox balance, regulated immune responses, metabolic reprogramming, and cellular homeostasis. Cross-omics-supported candidate factors (e.g., *P4ha1*, COX6B, RAB5A, *CAV3*) represent promising targets for functional validation via DNA-level experiments in independent sample sets, and the prominent enrichment of arginine-proline metabolism indicates a potential target for dietary intervention that merits further investigation.

## 1. Introduction

The large yellow croaker (*Larimichthys crocea*) is one of the most economically important marine fish species in China, with an annual aquaculture production of approximately 280,000 tons, ranking first among marine-cultured fish species nationwide [1]. However, intensive net-cage aquaculture practices and deterioration of coastal environments have led to frequent disease outbreaks in cultured stocks. Among these diseases, visceral white-nodules disease (VWND) caused by *Pseudomonas plecoglossicida* is especially devastating, with mortality rates exceeding 80% in affected populations [2,3].

*P. plecoglossicida* is a Gram-negative, flagellated opportunistic pathogen that invades the host *L. crocea* via epidermal wounds or mucosal surfaces and secretes a repertoire of extracellular proteases to compromise host tissue integrity [4,5]. The bacterium exhibits temperature-dependent virulence, with outbreaks typically occurring at 15–20 °C, a thermal window that overlaps with both the market-sized fish harvesting period and broodstock conditioning phase of *L. crocea* [3]. At the tissue level, *P. plecoglossicida* infection induces severe pathological damage to the liver, spleen and kidneys, with typical white granulomas formed in these organs [2]. In particular, histopathological examinations have revealed cell disintegration and substantial hemosiderin deposition in the liver of infected fish, confirming that the liver is a primary target organ of this pathogen [6]. This marked hepatic tropism implies that the liver plays a pivotal role in the host’s anti-*P. plecoglossicida* defense, yet the underlying molecular mechanisms remain largely uncharacterized.

The liver is a central metabolic and immune organ, playing a key role in nutrient metabolism, detoxification, and innate immunity in mammals [7]. In teleosts, the liver is similarly recognized as an important immune tissue, harboring diverse immune cells and expressing a wide array of immune-related genes [8]. For instance, the liver-expressed antimicrobial peptide 2 in the black rockfish exhibits potent antibacterial activity and is significantly upregulated upon bacterial challenge [9], and Collectin-11 in the obscure puffer activates the NF-κB pathway to mediate antibacterial immunity [10]. However, unlike the spleen and head kidney—which primarily serve as sites for immune initiation and hematopoiesis—the liver uniquely couples immune surveillance with systemic metabolic regulation, a distinction that makes immunometabolic reprogramming a defining feature of its defensive responses during infection [11,12]. This functional duality is particularly critical for *P. plecoglossicida* pathogenesis, as this pathogen preferentially colonizes the liver, induces extensive granuloma formation, and directly disrupts hepatic immunometabolic homeostasis via its T6SS effector vgrG1a by activating pro-inflammatory cascades while suppressing AMPK-mediated energy metabolism [6]. Despite these pathological and functional clues, the detailed molecular events orchestrating hepatic resistance in *L. crocea* against *P. plecoglossicida* remain largely unexplored.

High-throughput sequencing technologies have revolutionized the study of host–pathogen interactions by enabling global profiling of gene and protein expression [13]. Transcriptomics provides a snapshot of the transcriptional landscape, revealing the activation of immune signaling pathways, metabolic reprogramming, and cellular stress responses during infection [14]. For example, transcriptomic analysis of the spleen and head kidney has identified key immune processes involved in the defense of *L. crocea* against *P. plecoglossicida* [15,16]. Similarly, integrated multi-omics studies in other teleosts have successfully uncovered immune-related genes and pathways in the liver following bacterial challenge, including complement and coagulation cascades, cytokine–cytokine receptor interaction, and metabolic reprogramming [17,18,19]. Proteomics, which directly measures protein abundance, offers complementary insights into the functional execution of immune responses [20,21]. Thus, combined transcriptomic and proteomic analyses provide a powerful approach to dissect the molecular basis of disease resistance, and such integrative studies have been increasingly applied in fish immunology research [22,23,24].

Despite accumulating evidence that the liver is a primary target organ of *P. plecoglossicida* in *L. crocea*, the molecular events underlying hepatic responses to infection remain largely unexplored. In this study, we aimed to systematically characterize the temporal dynamics of liver pathology, oxidative stress, and the associated transcriptomic and proteomic landscapes following *P. plecoglossicida* infection. Histopathological examination and oxidative stress assays were performed to assess the progression of liver injury. Transcriptomic and proteomic profiling were conducted to map global changes in gene and protein expression over the course of infection. Through KEGG pathway enrichment analysis, we further integrated the two omics layers to identify core pathways consistently altered at both mRNA and protein levels. Our findings provide a detailed molecular framework for understanding liver defense mechanisms against bacterial infection and may inform the development of strategies for VWND control in *L. crocea* aquaculture.

## 2. Results

### 2.1. Survival, Phenotypic Distribution, and Liver Bacterial Load

**Figure 1:**
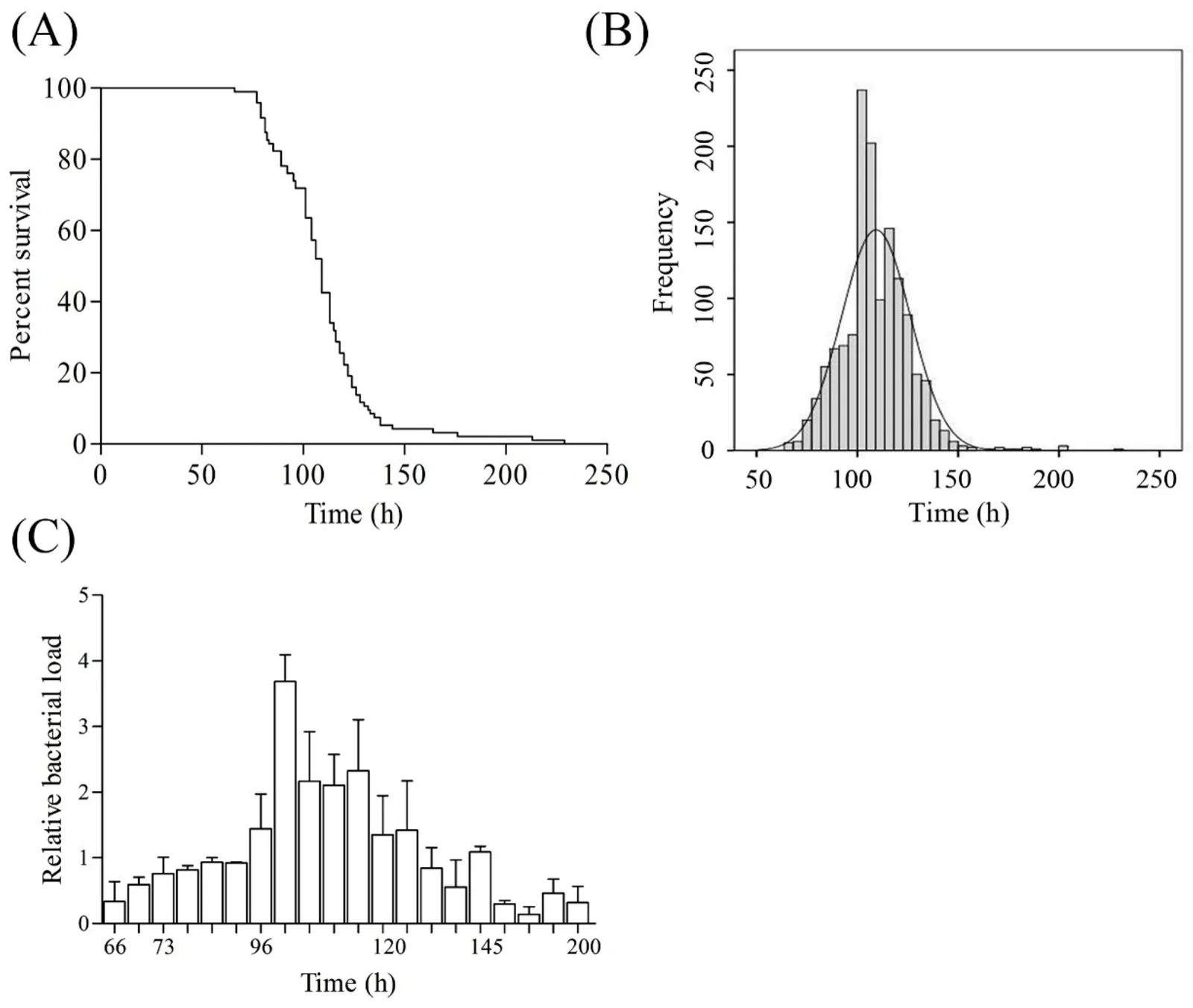
Phenotypes of the *L. crocea* after infection with *P. plecoglossicida*. (**A**) Survival curves of 1500 *L. crocea* after infected. (**B**) Frequency distribution of survival times post-infection. (**C**) Relative bacterial load in the liver of post-infection at different survival time points.

The survival curve of 1500 *L. crocea* after artificial infection is shown in Figure 1A. Mortality began 66 h post-infection, increased markedly thereafter, and ceased around 236 h. The frequency distribution of individual survival times (Figure 1B) exhibited a unimodal and approximately normal distribution, consistent with the characteristics of a quantitative trait. The relative bacterial load in the liver was measured at different survival time points (Figure 1C), and its distribution pattern was highly similar to that of survival time. Based on these results, individuals with extreme survival times were selected as the susceptible group (short survival) and the resistant group (long survival) for subsequent analyses.

### 2.2. Histopathological Alterations in the Liver

**Figure 2:**
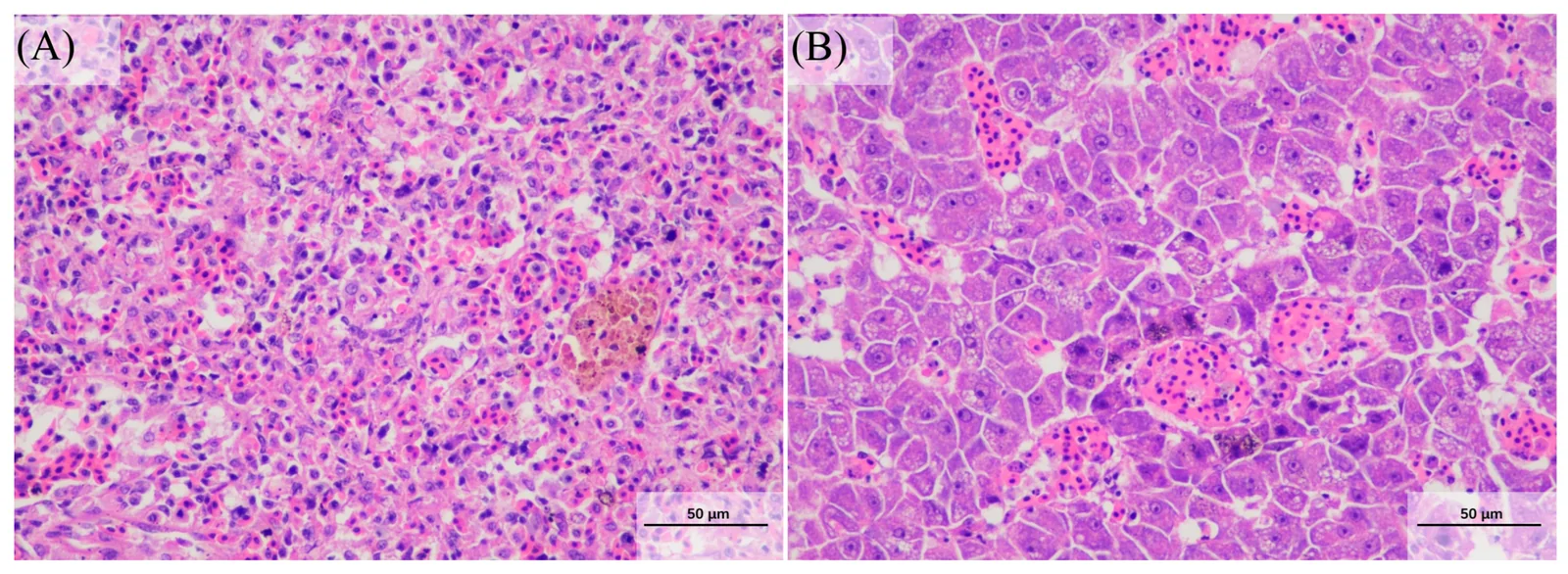
H&E-stained liver sections of *L. crocea* after infection. (**A**) Susceptible group: Widespread hepatocellular necrosis, vacuolar degeneration, inflammatory infiltration, and hemosiderin deposition. (**B**) Resistant group: Intact hepatic architecture with no obvious necrosis. Original magnification, ×400, *n* = 3.

H&E-stained liver sections from susceptible and resistant fish are shown in Figure 2. In the susceptible group (Figure 2A), the liver exhibited widespread hepatocellular necrosis, diffuse cytoplasmic vacuolation, abundant inflammatory cell infiltration, and numerous brownish-yellow granular deposits, consistent with hemosiderin. In the resistant group (Figure 2B), the liver showed markedly milder alterations, including mild to moderate vacuolation in scattered areas, occasional single-cell necrosis, and a few small inflammatory foci, while the overall hepatic architecture remained largely intact. No significant hemosiderin deposition was observed in the resistant group.

### 2.3. Liver Oxidative Stress Parameters

**Figure 3:**
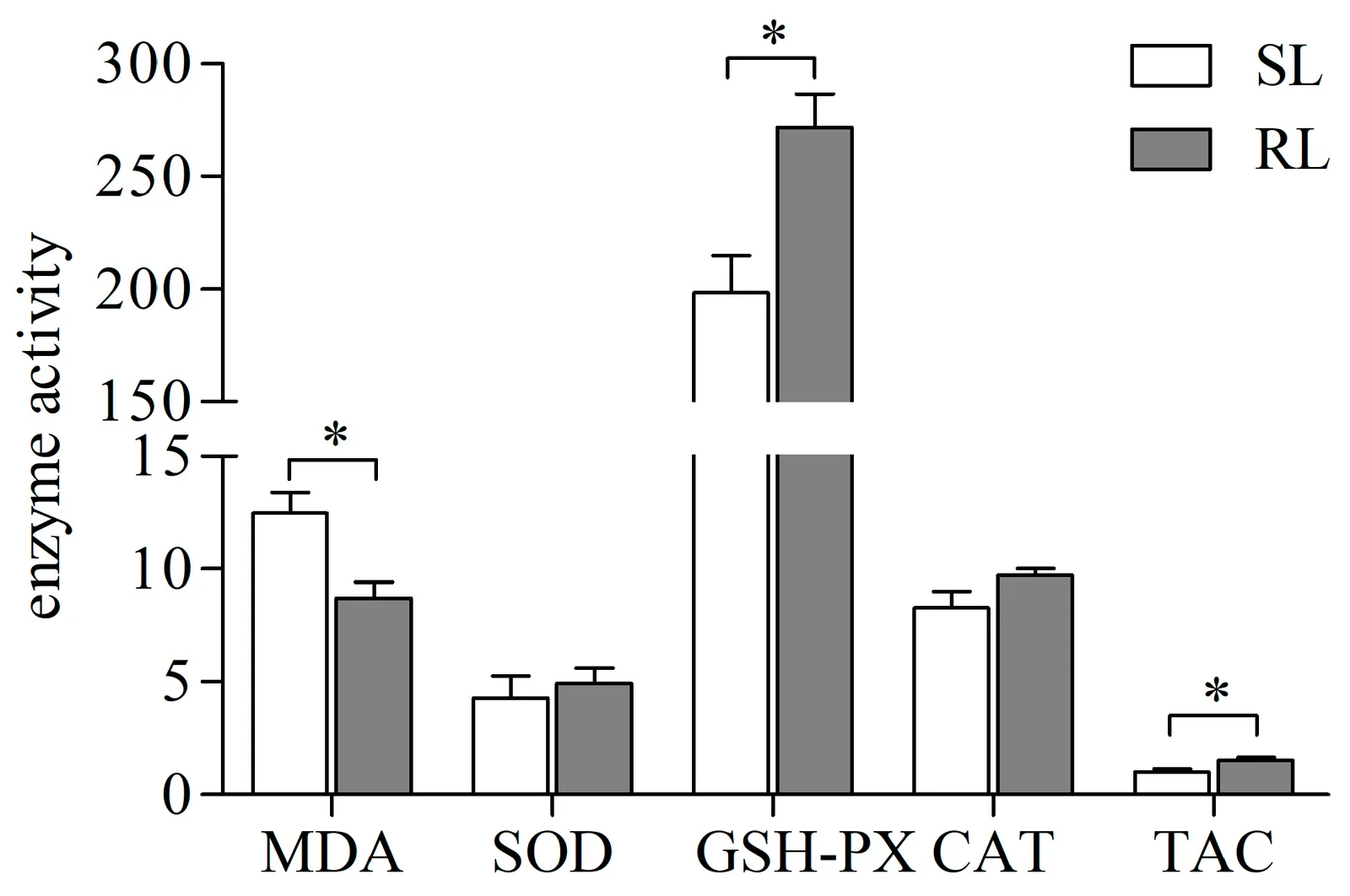
Oxidative stress indicators in the liver of susceptible (SL) and resistant (RL) fish after infection. Malondialdehyde (MDA, nmol/L). Superoxide dismutase (SOD, U/mL). Glutathione peroxidase (GSH-Px, U/L). Catalase (CAT, U/mL). Total antioxidant capacity (TAC, μmol Trolox/g fresh weight). Data are presented as mean ± SD (*n* = 3). “*” indicate significant differences between groups (*p* < 0.05).

The levels of oxidative stress markers in the liver are shown in Figure 3. Compared with the susceptible group, the resistant group exhibited significantly lower MDA content and significantly higher GSH-Px and TAC activities (*p* < 0.05). No significant differences were observed in SOD or CAT activities between the two groups (*p* > 0.05).

### 2.4. Transcriptomic Analysis of the Liver Between Susceptible and Resistant Groups

**Figure 4:**
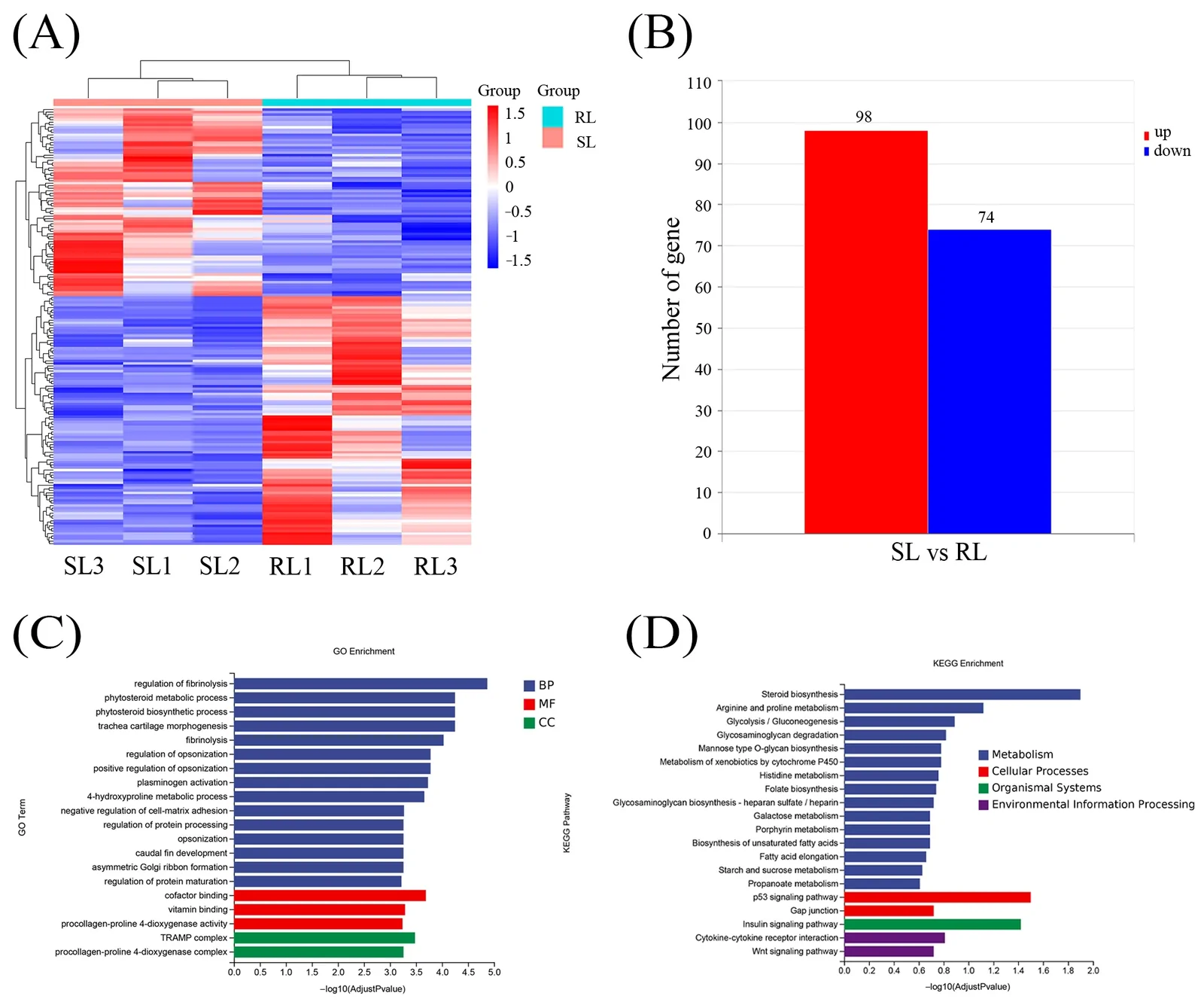
Transcriptomic analysis of the liver between susceptible (SL) and resistant (RL) groups. (**A**) Hierarchical clustering heatmap of gene expression profiles in SL and RLsamples (*n* = 3). Red and blue indicate high and low expression levels, respectively. (**B**) Number of DEGs between SL and RL groups. (**C**) GO enrichment analysis of DEGs. Selected enriched terms in biological process (BP), molecular function (MF), and cellular component (CC) are shown. (**D**) KEGG enrichment analysis of DEGs. The horizontal axis represents −log10(AdjustPvalue).

RNA sequencing was performed on liver samples from the SL and RL groups (PRJCA062549). Each sample generated 40.3–48.4 million clean reads (Supplementary Table S1), with Q30 values exceeding 93% and an average genome mapping rate of >90%, indicating high-quality data suitable for downstream analysis. Hierarchical clustering analysis (Figure 4A) clearly separated the SL and RL samples, indicating distinct gene expression patterns between the two groups. A total of 172 differentially expressed genes (DEGs) were identified between the two groups, among which 98 were significantly upregulated and 74 were downregulated in the RL group compared with the SL group (Figure 4B). GO enrichment analysis of the DEGs (Figure 4C) revealed that the most significantly enriched biological processes included regulation of fibrinolysis, ossification, and plasminogen activation, while enriched molecular functions included cofactor binding and procollagen-proline dioxygenase activity. KEGG pathway enrichment analysis (Figure 4D) showed that the DEGs were significantly enriched in pathways related to glycosaminoglycan biosynthesis, galactose metabolism, fatty acid elongation, and the p53 signaling pathway, as well as immune-related pathways such as cytokine–cytokine receptor interaction and the Wnt signaling pathway.

### 2.5. Proteomic Analysis of the Liver Between Susceptible and Resistant Groups

**Figure 5:**
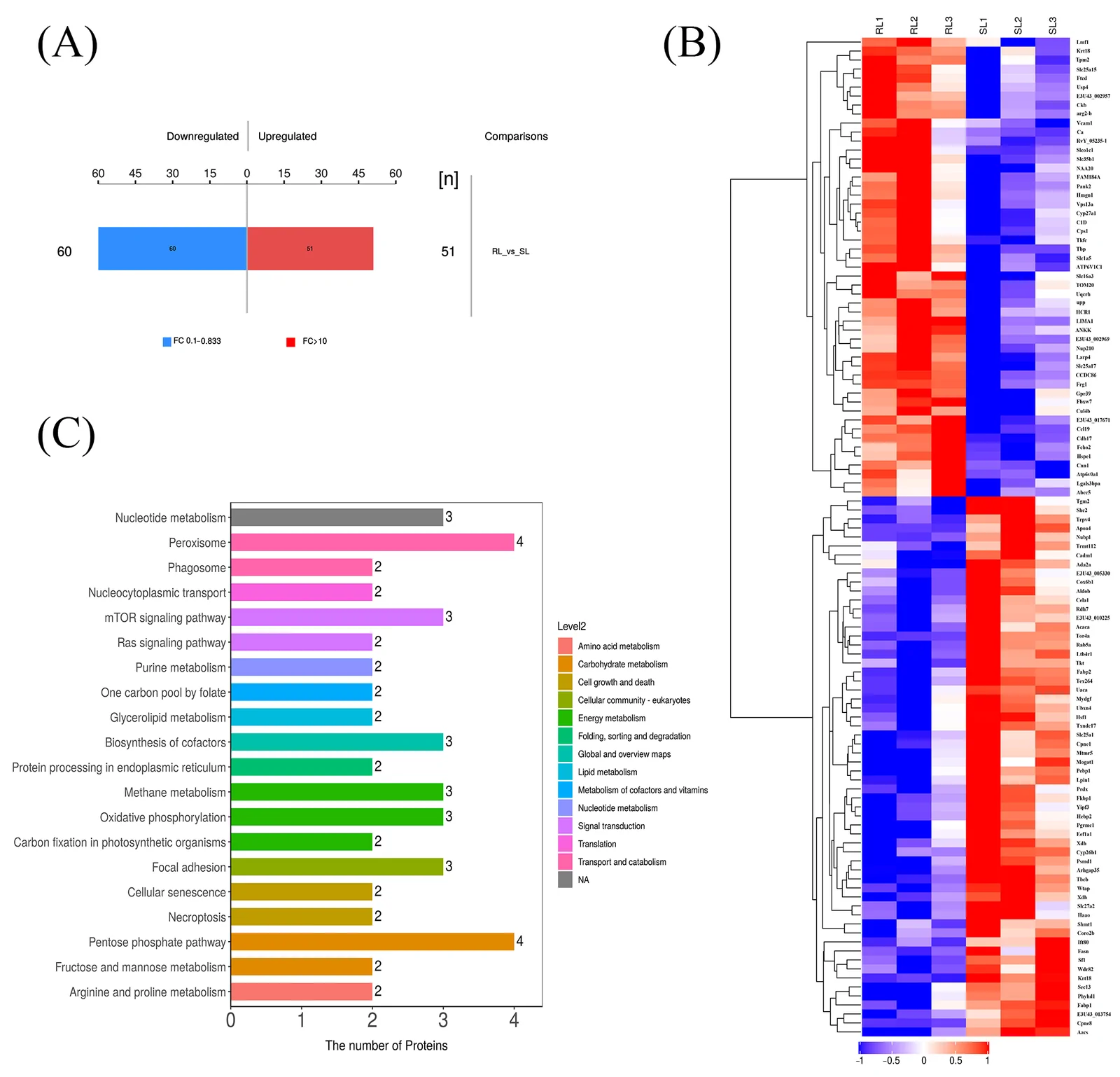
Proteomic analysis of the liver between SL and RL groups (*n* = 3). (**A**) Distribution of fold change (RL/SL) of DEPs. Blue bars: downregulated in RL (FC < 0.833); red bars: upregulated in RL (FC > 1.2). Darker shades indicate extreme changes (FC < 0.1 or FC > 10). Numbers above bars indicate DEP counts. (**B**) Total number of DEPs. (**C**) KEGG pathway enrichment analysis of DEPs. The y-axis lists selected enriched pathways, and the x-axis shows −log_10_(*p*-value). Pathways are grouped by functional categories (Level 2). Pathways with FDR < 0.05 (Benjamini–Hochberg adjusted) were considered significantly enriched.

Quantitative proteomic analysis using TMT labeling identified a total of 2599 quantifiable proteins in the liver of SL and RL groups (PRJCA062551). A total of 111 DEPs were identified between the two groups based on the criteria of fold change >1.2 (or <0.83) and *p* < 0.05 (Figure 5B). Among these, 51 proteins were significantly upregulated and 60 were downregulated in the RL group compared with the SL group. Further examination of the fold change distribution (Figure 5A) revealed that a notable proportion of DEPs exhibited extreme expression changes: among the downregulated proteins, 45 showed a fold change <0.1, and among the upregulated proteins, 15 showed a fold change >10.

KEGG pathway enrichment analysis was performed on the 111 DEPs to identify biological pathways potentially involved in resistance to *P. plecoglossicida* infection. As shown in Figure 5C, the DEPs were significantly enriched in several metabolic and cellular pathways. The most highly enriched pathway was the pentose phosphate pathway, followed by methane metabolism, peroxisome, one carbon pool by folate, and oxidative phosphorylation. Other enriched pathways included nucleotide metabolism, glycerolipid metabolism, protein processing in the endoplasmic reticulum, and focal adhesion. Notably, the peroxisome pathway, which is involved in reactive oxygen species (ROS) metabolism and fatty acid oxidation, was among the top enriched pathways, consistent with the observed differences in oxidative stress parameters between SL and RL groups (Figure 3). In addition, enrichment of the pentose phosphate pathway and one carbon pool by folate suggests alterations in cellular redox balance and one-carbon metabolism.

### 2.6. Integrated Analysis of Multi‑Omics Data Between Susceptible and Resistant Groups

To assess the overall consistency between the two omics datasets, Spearman correlation analysis of all corresponding gene–protein pairs yielded a correlation coefficient of 0.31. Among the 111 DEPs, 32 showed concordant directional changes with their corresponding transcripts (17 upregulated and 15 downregulated at both mRNA and protein levels), while the remaining 79 DEPs exhibited discordant patterns. Functional enrichment analysis of the discordant gene-protein pairs revealed pathways related to proteolysis, amino acid metabolism, and ECM-receptor interaction (Supplementary Figure S1).

A STRING-based protein–protein interaction (PPI) network was constructed for genes annotated in these pathways. The resulting network revealed limited cross-pathway connectivity, with most nodes remaining isolated and only a few direct edges identified (Supplementary Figure S2), suggesting that these resistance-associated pathways function as relatively independent modules.

Accordingly, we focused on the 8 KEGG pathways that were co-enriched at both transcriptomic and proteomic levels between resistant (RL) and susceptible (SL) individuals (defined as pathways with FDR < 0.05 in both omics layers) (Table 1). These pathways were mainly distributed in amino acid metabolism, immune signaling, cell death, signal transduction, energy metabolism, carbohydrate metabolism, immune clearance, and cell adhesion, representing the core functional modules underlying hepatic resistance against *P. plecoglossicida*. Notably, most key genes and proteins in these pathways exhibited consistently upregulated expression in resistant fish, including those involved in arginine and proline metabolism (*P4ha1*, *Hoga1*, Arginase), immune signaling (*Epo*, *BMPR1B*, CCL19), energy supply (COX6B, QCR6, ALDO), phagocytic pathogen clearance (RAB5A, V-ATPase), and hepatic structure maintenance (*CAV3*, CAPN2, ARHGAP35). These convergent molecular changes at mRNA and protein levels collectively indicated that resistant individuals possessed a strengthened capacity for antibacterial defense, inflammatory homeostasis, energy provision, moderate apoptosis, efficient pathogen clearance, and liver structural stability, which may directly determine the survival advantage of resistant fish.

### 2.7. Validation of DEGs with RT-qPCR

**Figure 6:**
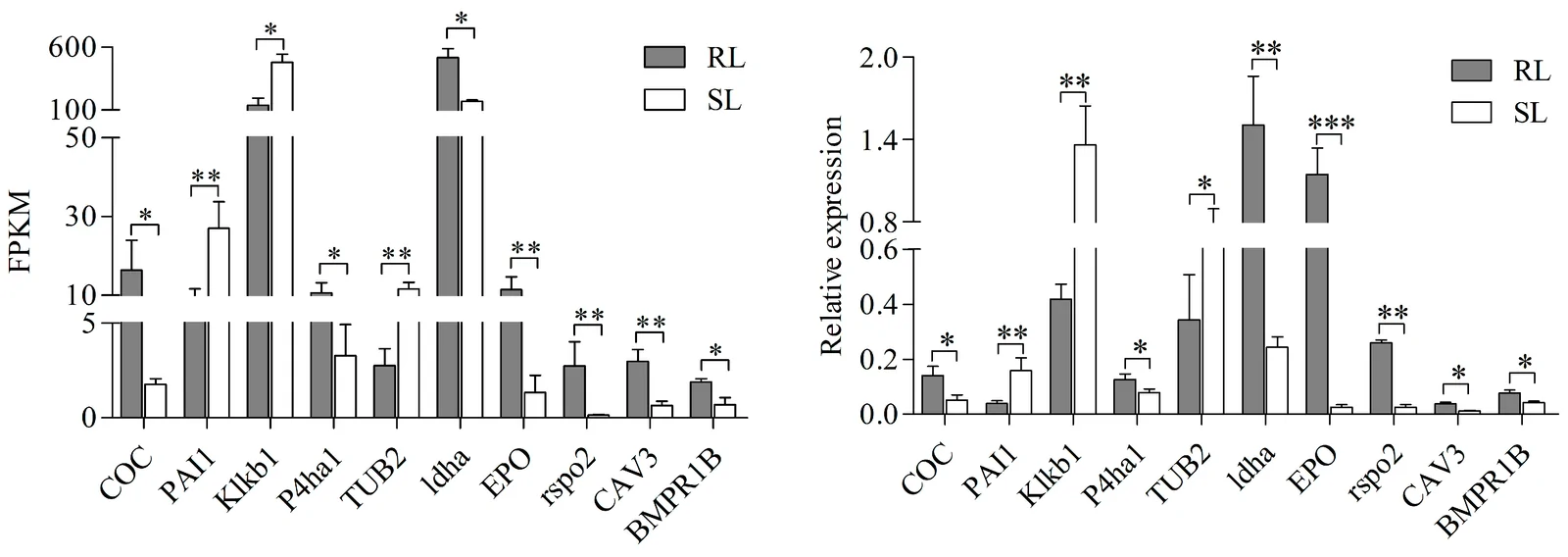
Validation of RNA-seq data by qPCR in the liver of SL and RL groups (*n* = 3). Left y-axis: FPKM values from RNA-seq (bars); right y-axis: relative mRNA expression levels from qPCR. Data are presented as mean ± SD. Asterisks indicate significant differences between SL and RL groups (“*” *p* < 0.05, “**” *p* < 0.01, “***” *p* < 0.001).

To validate the reliability of the transcriptomic data, 10 DEGs were selected for qPCR analysis in the liver of SL and RL groups (Figure 6). The qPCR results show that the expression levels of *COC*, *P4ha1*, *Ldh-a*, *Epo*, *CAV3*, and *BMPR1B* were significantly higher in the RL group than in the SL group (*p* < 0.05), whereas the expression levels of *PAI1*, *K1kb1*, and *TUB2* were significantly lower in the RL group (*p* < 0.05). The direction of differential expression for all tested genes was consistent with that observed in the RNA-seq data (Figure 6), confirming the reliability of the transcriptomic profiles.

## 3. Discussion

### 3.1. Rigorous Phenotypic Classification Ensures the Validity of Extreme Phenotype Materials

Identifying disease-resistant and susceptible individuals represents the first and most critical step toward dissecting the underlying molecular mechanisms of disease resistance. In this study, we developed a dual-indicator phenotyping system that integrates survival time and hepatic bacterial load to classify 1500 *L. crocea* individuals following *P. plecoglossicida* infection. The unimodal, approximately normal distribution of survival times confirmed that VWND resistance is a quantitative trait, which aligns with prior findings on disease resistance in *L. crocea* [25,26]. As a typical polygenic trait controlled by multiple genes of small to moderate effect, quantitative disease resistance makes extreme phenotype selection a powerful strategy to enrich resistance-associated genetic variants [27,28].

The incorporation of liver pathogen load as an auxiliary phenotyping indicator further improved the accuracy of phenotype stratification. Our results revealed a unimodal trend in hepatic bacterial load along the survival time spectrum: levels first rose and then fell as survival time increased, with both disease-resistant and susceptible groups exhibiting low hepatic bacterial loads, while individuals with intermediate survival times harbored significantly higher pathogen burdens. This pattern indicates that resistant individuals are not simply postponing mortality but are actively curbing hepatic pathogen proliferation—a core function of the liver, a critical immune-metabolic organ in teleosts [29]. By selecting the 30 shortest-survival and 30 longest-survival individuals from the tails of the distribution, we effectively minimized the interference of inter-individual genetic and environmental heterogeneity, ensuring that the subsequent multi-omics comparisons would capture signals truly associated with resistance rather than random noise. This extreme phenotype selection strategy is widely applied in aquaculture disease resistance research, including bulked segregant analysis (BSA)-seq and transcriptomic profiling in *L. crocea* and other species [7,11,30].

### 3.2. Histopathological and Oxidative Stress Differences Confirm the Micro-Level Divergence Between SL and RL

The liver is the central immune-metabolic organ of teleosts and the primary target of *P. plecoglossicida* infection [24]. Histopathological examination revealed that susceptible fish exhibited severe liver injury, including extensive hepatocellular necrosis, inflammatory infiltrates, and hemosiderin deposition, whereas resistant individuals retained an intact liver architecture with only mild vacuolar degeneration. This stark contrast in liver tissue integrity directly mirrors host resistance levels, as structural preservation is essential for sustaining normal immune and metabolic functions [31].

Oxidative-stress biomarkers further discriminated the two phenotypes. Resistant fish displayed significantly lower MDA content (reduced lipid peroxidation) and significantly higher GSH-Px activity and TAC than susceptible counterparts, reflecting a more robust glutathione-dependent antioxidant defense. Because the liver possesses a high metabolic rate and abundant polyunsaturated fatty acids, it is especially prone to oxidative insult during bacterial infection [32]. Infection with *P. plecoglossicida* triggers reactive oxygen species (ROS) production as part of the innate immune response; however, excessive ROS can inflict collateral tissue damage [33]. The elevated GSH-Px and TAC observed in resistant fish suggest a superior capacity to scavenge ROS and preserve redox homeostasis, thereby limiting infection-induced liver injury. Comparable patterns have been reported in other teleosts facing bacterial challenge, where resistant individuals typically exhibit higher antioxidant enzyme activities and lower lipid peroxidation [34,35]. Notably, SOD and CAT activities did not differ significantly between resistant and susceptible groups, which may reflect temporal dynamics in antioxidant enzyme regulation. Unlike GSH-Px, which is induced by sustained oxidative stress, SOD and CAT are largely constitutively expressed and primarily modulated post-translationally [36]. Sampling at the time of death may therefore capture a phase when SOD and CAT activities have returned to baseline, whereas GSH-Px remains elevated due to ongoing lipid peroxidation [32]. Nevertheless, the combination of reduced MDA, elevated GSH-Px, and heightened TAC in resistant fish supports an enhanced glutathione-dependent antioxidant capacity.

Taken together, the histopathological and oxidative stress results confirm that the extreme phenotypic classification we devised corresponds to meaningful cellular differences between susceptible and resistant fish. These distinctions validate the resistant group as a bona fide “resistant” phenotype, providing a solid foundation for downstream transcriptomic and proteomic analyses. At the tissue level, the findings indicate that the liver—a primary target of *P. plecoglossicida* [2,6]—harbors resistance-associated pathological and redox signatures, implicating hepatic factors in host defense. In teleosts, the liver functions as the nexus of systemic metabolism and immune regulation [29], offering a perspective distinct from that of the spleen and head kidney. Consequently, it is uniquely suited for investigating the immunometabolic reprogramming and systemic inflammatory coordination that may underlie disease resistance.

### 3.3. Transcriptomic Signatures of a Balanced Immune Response

Transcriptomic analysis identified 172 DEGs between RL and SL. KEGG enrichment revealed that cytokine–cytokine receptor interaction, Wnt signaling, glycolysis/gluconeogenesis and p53 signaling were among the most significantly enriched pathways. These pathways have been implicated in host defense against bacterial infection in teleosts. Cytokine–cytokine receptor interaction is central to orchestrating inflammatory and adaptive immune responses [36]; Wnt signaling regulates tissue repair and immune homeostasis [37]; glycolysis/gluconeogenesis supports the metabolic shift required for activated immune cells [38]; and p53 signaling regulates apoptosis and cellular stress responses, which are essential for clearing infected hepatocytes while avoiding widespread necrosis [39], aligning with the severe hepatic injury observed in susceptible individuals in our histopathological analysis.

Within these pathways, several key genes exhibited opposite expression patterns in RL, pointing to a finely tuned rather than uniformly activated immune state. *Epo* was significantly upregulated in RL. Beyond its classical role in erythropoiesis, *Epo* possesses immunomodulatory and tissue-protective functions. In mammalian models, *Epo* suppresses pro-inflammatory cytokine production and limits tissue damage during bacterial infection [40,41]. Teleost *Epo* homologs are responsive to immune challenge [42], suggesting that resistant fish may employ *Epo*-mediated anti-inflammatory mechanisms to limit hepatic injury. Conversely, *PAI1* and *K1kb1* were significantly downregulated in RL. *PAI1* is a major inhibitor of fibrinolysis; its downregulation promotes fibrinolytic activity, preventing excessive thrombosis and maintaining microcirculation during sepsis [43]. *K1kb1* is a key component of the contact activation system linking coagulation and inflammation; its downregulation may suppress excessive inflammatory and coagulation cascades, thereby protecting the liver from immunopathology [44]. These expression patterns align with the GO enrichment of “regulation of fibrinolysis” and “plasminogen activation”. In addition, *CAV3* was upregulated in RL. Caveolae function as signaling hubs involved in endocytosis and immune regulation; in teleosts, caveolin-1 has been implicated in bacterial infection [45]. The upregulation of *CAV3* may contribute to maintaining membrane integrity and coordinating immune signaling.

Collectively, the transcriptomic profile of RL is defined by simultaneous upregulation of anti-inflammatory and tissue-protective genes (*Epo*, *CAV3*) and downregulation of pro-coagulant and pro-inflammatory factors (*PAI1*, *K1kb1*). This distinct expression signature suggests that resistant fish may achieve a balanced immune response, sufficient to control pathogen proliferation, yet restrained to avoid excessive inflammation and tissue damage—a hallmark of effective disease resistance [46]. These transcriptomic signatures are consistent with the attenuated hepatic injury and enhanced antioxidant capacity observed in RL, providing mechanistic insights into the resistance phenotype and laying a foundation for subsequent multi-omics integration analyses.

### 3.4. Proteomic Evidence for Enhanced Metabolic and Phagocytic Capacity

Proteomic analysis identified 111 DEPs between RL and SL. KEGG enrichment revealed that pathways related to peroxisome, oxidative phosphorylation, pentose phosphate pathway, and phagosome were significantly enriched in RL relative to SL. Collectively, these pathways are well-documented to play critical roles in host defense against bacterial infection in teleosts. Peroxisomes are central to cellular redox homeostasis; they generate and scavenge ROS, and their dysfunction exacerbates infection-induced oxidative injury [47]. The enrichment of peroxisome pathway in RL aligns with the observed lower MDA and higher TAC in resistant fish, suggesting that RL fish possess an enhanced capacity to neutralize ROS and limit lipid peroxidation. The pentose phosphate pathway generates NADPH, which is essential for both antioxidant defense (via glutathione regeneration) and phagocyte oxidative burst [48]. The enrichment of this pathway in RL further supports the notion that resistant fish upregulate metabolic programs that underpin redox balance and effective immune function.

The phagosome pathway was also significantly enriched in RL, with upregulated key proteins including RAB5A and V-ATPase. RAB5A regulates early endosome fusion and phagosome maturation, and its upregulation has been linked to improved bacterial clearance in macrophages [49]. V-ATPase is responsible for phagosomal acidification, a critical step for killing and degrading engulfed pathogens [50]. Thus, the proteomic data indicate that resistant fish enhance their phagocytic capacity at the protein level, which likely contributes to the reduced hepatic pathogen load. Notably, a transcript-protein discordance was observed for phagosome-related molecules: while the transcriptome showed downregulation of *TUB2* (a phagosome-related gene), the proteome exhibited upregulation of RAB5A and V-ATPase. Such gene-protein discordance is common in host–pathogen interactions, where post-transcriptional regulation (e.g., translation efficiency, protein stability) can uncouple mRNA abundance from protein function [18]. This observation underscores the value of integrating proteomics with transcriptomics to capture functionally relevant changes that may be missed at the mRNA level.

### 3.5. Integrated Pathway Analysis Identifies Core Resistance Modules

Comparative analysis of KEGG enrichment results from transcriptomic and proteomic profiling identified 8 pathways consistently co-enriched across both omics layers. These pathways fall into six functional categories: amino acid metabolism (arginine and proline), immune signaling (cytokine–cytokine receptor interaction), cell death (apoptosis), signal transduction (mTOR), energy metabolism (oxidative phosphorylation, glycolysis/gluconeogenesis), immune clearance (phagosome), and cell adhesion (focal adhesion). The consistent enrichment of these pathways at both mRNA and protein levels supports their functional relevance to resistance. Among these pathways, the arginine and proline metabolism pathway is particularly notable. Arginine is a substrate for both inducible nitric oxide synthase (iNOS) and arginase: iNOS catalyzes the production of nitric oxide (NO), a potent antimicrobial effector molecule, while arginase produces polyamines and proline, which promote tissue repair and cell proliferation [51]. In resistant individuals, the upregulation of *P4ha1* (at the transcript level) and arginase/creatine kinase (at the protein level) indicates a metabolic shift toward arginase-mediated pathways, favoring tissue repair and anti-inflammatory responses over excessive NO production. This metabolic shift may protect the liver from NO-induced cytotoxicity while still allowing some antimicrobial activity. A similar arginase-biased response has been observed in other fish species during bacterial infection and is associated with improved survival [12,52]. Additionally, the co-enrichment of oxidative phosphorylation and glycolysis/gluconeogenesis indicates that resistant fish reprogram their energy metabolism to meet the increased demands of immune activation. Activated immune cells undergo a metabolic switch from oxidative phosphorylation to aerobic glycolysis to rapidly generate ATP and biosynthetic precursors [38]. Notably, the simultaneous upregulation of both pathways in RL suggests that resistant fish are capable of supporting both the rapid glycolytic burst required for immediate immune responses and the sustained oxidative phosphorylation needed for long-term immune homeostasis. This metabolic flexibility is a hallmark of effective immunity and has been documented in teleosts under pathogen challenge [53].

Further, the apoptosis pathway, with upregulation of *COX* and *calpain-2*, suggests that resistant fish maintain moderate apoptosis to eliminate infected hepatocytes while preventing widespread necrosis—a protective mechanism that limits inflammation [54]. The mTOR signaling pathway coordinates energy metabolism and autophagy, and its enrichment (with upregulation of LPIN, V-ATPase, SEC13) indicates sustained homeostatic control during infection [55]. The focal adhesion pathway, with upregulation of CAPN2, ARHGAP35 and SHC2, enhances cell–cell junctions and maintains liver structural integrity, which likely underlies the mild histopathological damage in resistant fish. Comparable multi-omics signatures of these pathways have been reported in *L. crocea* selectively bred for VWND resistance [56] and in rainbow trout selected for bacterial cold-water disease resistance [57]. These pathways operate across distinct functional tiers, a pattern consistent with our protein–protein interaction (PPI) analysis, which showed limited cross-pathway connectivity and a modular organization of DEPs. Notably, this pattern differs from observations in spleen and head kidney, where DEGs are typically enriched in canonical immune pathways including pathogen recognition, inflammatory regulation, and T-cell activation [11,12,16]. As a multifunctional metabolic and detoxification organ, the liver likely coordinates host resistance via multiple parallel layers, integrating immune, metabolic, and structural protective processes [22].

Beyond these concordant changes observed across both omics layers, discordant gene–protein pairs (i.e., protein abundance changes not mirrored at the transcript level) were significantly enriched in pathways related to proteolysis, amino acid metabolism, and extracellular matrix (ECM)–receptor interaction. This pattern suggests that post-translational regulation of proteolytic processes and ECM remodeling may also play important roles in the hepatic response to *P. plecoglossicida* infection, potentially modulating hepatic tissue integrity and immune cell infiltration into the liver parenchyma. While the functional significance of these discordant regulatory patterns requires further experimental validation, they highlight the complementary value of integrated multi-omics analysis: proteomic data can capture post-transcriptional regulatory layers that are not detectable via transcriptomic profiling alone.

### 3.6. Limitations and Future Directions

This study represents an important step toward understanding the molecular basis of resistance to *P. plecoglossicida* in *L. crocea* through integrated multi-omics profiling of extreme phenotypes. Nevertheless, several limitations warrant consideration. First, the identified pathways and candidate genes are correlative rather than causal; functional validation—such as dietary arginine supplementation or gene knockdown/overexpression assays—is required. Second, phenotypic classification was based on survival time and bacterial load assessed at death, longitudinal sampling would provide deeper insights into the temporal dynamics of resistance. Third, the sample size for omics analyses, while sufficient for differential expression detection, may limit the power to detect subtle changes and should be expanded in future studies. Fourth, the present investigation focused exclusively on the liver, although this choice was justified by its role as the primary target organ of *P. plecoglossicida*, resistance mechanisms operating in other immune organs (spleen and head kidney) remain unexplored. Fifth, an uninfected control group was included in the challenge experiment but was not subjected to multi-omics analysis; therefore, the current findings reflect differences between resistant and susceptible infected individuals rather than infection-specific transcriptional or proteomic responses. Despite these limitations, the multi-omics dataset presented here provides a valuable resource and a focused set of hypotheses for future mechanistic investigations in this economically important species.

## 4. Materials and Methods

### 4.1. Experimental Fish and Bacterial Challenge

The *L. crocea* used in this study were second-generation offspring of the Daiqu strain wild population. A total of 1500 healthy individuals (body length: 15.3 ± 2.2 cm; body weight: 36.5 ± 7.8 g) were randomly selected from the same cultured population and acclimated in laboratory tanks for two weeks prior to the infection experiment. Artificial infection was performed following our previously established protocol [11]. Prior to injection, fish were anesthetized with MS-222 (50 mg/L). Briefly, each fish was intraperitoneally injected with 0.5 mL of a bacterial suspension of *P. plecoglossicida* (XSDHY-P strain) at a concentration of 1 × 10^5^ CFU/mL. Fish in the control group (*n* = 30) received an equal volume of sterile culture medium. After injection, the fish were evenly distributed into ten 2.5-ton polyethylene tanks at a density of 150 fish per tank. Throughout the experimental period, fish were continuously monitored, and mortality was recorded. Fish were considered dead when unresponsive to external stimuli. Dead individuals were promptly removed, and their survival time was recorded. For all 1500 fish, liver tissues were immediately collected after death, frozen in liquid nitrogen in 2 mL sterile tubes, and stored at −80 °C for subsequent analyses.

### 4.2. Phenotyping Disease Resistance

Survival time (AT) after infection was recorded for each individual and used as the primary criterion to classify susceptible and resistant groups. To further characterize the resistance phenotype, the relative abundance of *P. plecoglossicida* in the liver was quantified as a supplementary indicator. Specifically, for extreme phenotype selection, the five shortest-surviving and five longest-surviving fish were first selected from each tank, yielding 50 candidates each for the susceptible and resistant groups. From these candidates, the 30 individuals with the highest liver bacterial load among the short-survival group were designated as the susceptible group (SL), while the 30 with the lowest bacterial load among the long-survival group were designated as the resistant group (RL). This two-step selection ensured that the SL group represented individuals with both rapid mortality and high pathogen proliferation, whereas the RL group represented individuals with prolonged survival and effective pathogen restriction.

Total DNA was extracted from the liver of all 1500 fish using the E.Z.N.A. Bacterial DNA Kit (Omega, Stamford, CT, USA) according to the manufacturer’s instructions. DNA integrity was assessed by 1% agarose gel electrophoresis, and DNA concentration and purity were measured using a spectrophotometer (NanoDrop, Thermo Fisher Scientific, Waltham, MA, USA). All DNA samples were diluted to a final concentration of 50 ng/μL. Quantitative real-time PCR (qPCR) was performed using specific primers for *P. plecoglossicida* (pps-F: GGTACTCACCTGGTTGGCTT; pps-R: GCTTGTCCTTGGTCTGGGAG). A single-copy gene of *L. crocea*, gdf8 (gdf8-F: TGGGAGATGACAACAGG; gdf8-R: GCACCGACCAATACACT), was used as an endogenous reference [11,58]. The relative abundance of *P. plecoglossicida* in liver cells was calculated using the 2^−ΔΔCt^ method. The qPCR program was set as follows: 95 °C for 1 min, followed by 40 cycles of 95 °C for 5 s, 60 °C for 10 s, and 72 °C for 15 s, with a final cooling step at 4 °C.

### 4.3. Histological Examination

Liver tissues from the SL and RL groups were used for histopathological observation. Approximately 0.5 cm of the liver tip was dissected and immediately fixed in 5 mL of 4% paraformaldehyde for 24 h at 4 °C. After fixation, the tissues were dehydrated through a graded ethanol series (70%, 80%, 90%, 95%, and 100%), cleared in xylene, and embedded in paraffin wax. Sections of 5 μm thickness were cut using a rotary microtome (Leica RM2255, Leica Biosystems, Nussloch, Germany) and mounted on glass slides. The sections were then deparaffinized, rehydrated, and stained with hematoxylin and eosin (H&E) following standard protocols. Finally, the stained sections were observed under a light microscope (Olympus BX53, Tokyo, Japan) at 400× magnification. Representative images were captured for histological assessment, focusing on hepatocellular necrosis, vacuolation, inflammatory infiltration, and hemosiderin deposition. Three individuals per group were examined.

### 4.4. Oxidative Stress Enzyme Activity Assays

Liver samples from the SL and RL groups were used to determine the activities of antioxidant enzymes and the content of malondialdehyde (MDA). For each group, nine individuals were randomly selected from the 30 extreme-phenotype fish. These nine individuals were equally divided into three pools (three fish per pool), each pool serving as one biological replicate (*n* = 3 per group). Approximately 50 mg of frozen liver tissue from each pool was homogenized in 0.5 mL of ice-cold phosphate-buffered saline (PBS, 0.1 M, pH 7.4) using a tissue homogenizer. The homogenate was centrifuged at 6000× *g* for 20 min at 4 °C, and the supernatant was collected for subsequent assays. Protein concentration was determined using the bicinchoninic acid (BCA) method with bovine serum albumin as the standard.

The activities of superoxide dismutase (SOD), glutathione peroxidase (GSH-Px), catalase (CAT), and total antioxidant capacity (TAC), as well as the MDA content, were measured using commercial kits (Nanjing Jiancheng Bioengineering Institute, Nanjing, China) according to the manufacturer’s instructions. For each group, three biological replicates (each pooled from three fish) were used for these assays, as described above. Briefly, SOD activity was measured using the nitroblue tetrazolium reduction inhibition assay with xanthine and xanthine oxidase as the superoxide generating system, and absorbance was recorded at 450 nm. GSH-Px activity was determined by the colorimetric method of Tietze at 405 nm. CAT activity was evaluated by monitoring the decomposition of H_2_O_2_ at 240 nm. TAC was assessed using the ferric reducing ability of plasma (FRAP) method at 593 nm. MDA content was measured using the thiobarbituric acid (TBA) method at 532 nm. All enzyme activities and MDA content were normalized to the total protein concentration in the supernatant. Each sample was measured in triplicate.

### 4.5. RNA Extraction and Transcriptome Sequencing

Liver tissues from the susceptible (SL) and resistant (RL) groups were used for transcriptome sequencing. For each group, nine individuals were randomly selected from the individually numbered pool of 30 extreme-phenotype fish and equally divided into three pools (three fish per pool). Pooling multiple individuals per replicate helps mitigate inter-individual variability, and the extreme-phenotype design further amplifies the resistance-associated signals. Then, each pool was homogenized and split into two aliquots: one for RNA extraction and transcriptomic sequencing, and the other for protein extraction and proteomic analysis, yielding three biological replicates per group for each omics layer. Total RNA was extracted from pooled liver tissue using the Total RNA Purification Kit (Omega, Mountain Lakes, NJ, USA) according to the manufacturer’s instructions. RNA integrity and concentration were assessed by 0.8% agarose gel electrophoresis and a NanoDrop 2000 spectrophotometer (Thermo Fisher Scientific, Waltham, MA, USA).

cDNA libraries were constructed and sequenced on the Illumina MiSeq platform (Personal Biotechnology Co., Ltd., Shanghai, China) following the standard protocol. Detailed sequencing statistics for each sample are provided in Supplementary Table S1. Raw reads were filtered to remove adapters and low-quality reads, and the resulting clean reads were aligned to the *L. crocea* reference genome (PRJNA1168280) using HISAT2 software (version 2.2.3). Gene expression levels were quantified as fragments per kilobase of transcript per million mapped reads (FPKM) using RSEM. Differentially expressed genes (DEGs) between the SL and RL groups were identified using DESeq2 (version 1.50.2) with the criteria of |log_2_(fold change)| ≥ 1 and *p* < 0.05.

### 4.6. Proteomic Analysis

Liver tissues from the susceptible (SL) and resistant (RL) groups were used for TMT-based quantitative proteomic analysis. Protein extraction, trypsin digestion using the FASP method, and TMT 6-plex labeling (Thermo Fisher Scientific, USA) were performed according to standard protocols using the same pooled samples described in Section 4.5. The labeled peptides were pooled and fractionated by high-pH reversed-phase chromatography into 10 fractions.

LC-MS/MS analysis was carried out on a Q-Exactive mass spectrometer coupled with an Easy nLC 1000 system (Thermo Fisher Scientific, USA). The mass spectrometer was operated in positive ion mode with a full scan range of 300–1800 *m*/*z* at a resolution of 70,000 (at *m*/*z* 200), with an AGC target of 1 × 10^6^ and a maximum injection time of 50 ms. The 20 most intense precursor ions were selected for MS/MS fragmentation using higher-energy collisional dissociation (HCD) with a normalized collision energy of 30 eV. MS/MS spectra were acquired at a resolution of 17,500 (at *m*/*z* 200) with an isolation window of 2 *m*/*z* and a dynamic exclusion time of 60 s. Raw data were searched against the *L. crocea* reference proteome database using Mascot 2.2 and Proteome Discoverer 1.4. Carbamidomethylation of cysteine and TMT 6-plex (lysine and peptide N-terminus) were set as fixed modifications, and oxidation of methionine as a variable modification. Peptide and protein identifications were filtered at a false discovery rate (FDR) ≤ 1%.

Protein quantification was based on the median of unique peptide ratios. Differentially expressed proteins (DEPs) were defined as those with a fold change >1.2 (or <0.83) and *p* < 0.05.

### 4.7. Integrated Analysis of Transcriptomic and Proteomic Data

To obtain a comprehensive understanding of the molecular mechanisms underlying *P. plecoglossicida* resistance in *L. crocea*, an integrated analysis of transcriptomic and proteomic data was performed. By comparing the proteome and transcriptome datasets, genes and proteins with one-to-one correspondence were obtained. Spearman correlation analysis was performed to evaluate the overall consistency between mRNA and protein expression fold changes (RL/SL), and the correlation between the two omics datasets was assessed based on the correlation coefficient. Genes and proteins with different change patterns (concordant or discordant) were subjected to functional enrichment analysis to explore the biological processes stably involved in resistance as well as those potentially associated with post-transcriptional regulation. For both omics datasets, multiple testing correction was performed using the Benjamini–Hochberg (BH) method to control the false discovery rate (FDR). Pathways with an FDR < 0.05 were considered significantly enriched.

### 4.8. Validation of Transcriptomic Data by qPCR

Ten differentially expressed genes were selected for qPCR validation using the same liver samples as for RNA-seq. First-strand cDNA was synthesized using EasyScript First-Strand cDNA Synthesis SuperMix (TransGen Biotech, Beijing, China). qPCR was performed with FastFire qPCR PreMix (SYBR Green) (Tiangen, Beijing, China) on a QIAquant 96 2plex instrument (QIAGEN, Hilden, Germany). The thermal cycling protocol was: 95 °C for 5 min, followed by 40 cycles of 95 °C for 15 s, 60 °C for 30 s, and 72 °C for 15 s. Relative expression levels were calculated using the 2^−ΔΔCt^ method with β-actin as the internal reference. The consistency of fold changes between qPCR and RNA-seq was evaluated. Primers are listed in Supplementary Table S2.

### 4.9. Statistical Analysis

All data are presented as mean ± standard deviation (SD). Normality of the data was assessed using the Kolmogorov–Smirnov test, and homogeneity of variances was tested using Levene’s test. For comparisons between two groups (SL vs. RL), an independent samples *t*-test was applied. A *p* < 0.05 was considered statistically significant, and *p* < 0.01 was considered highly significant. All statistical analyses were performed using SPSS version 19.0 (IBM, Armonk, NY, USA). Graphs were generated using GraphPad Prism version 5.0 (GraphPad Software, San Diego, CA, USA).

## 5. Conclusions

This study provides a multi-omics dissection of liver resistance mechanisms in *L. crocea* against *P. plecoglossicida*. By selecting extreme phenotypes based on survival time and liver pathogen load, we found that resistant fish preserve liver architecture, display heightened antioxidant defenses (lower MDA, higher GSH-Px/TAC), and mount a balanced immune response that avoids excessive inflammation. Integrated transcriptomic and proteomic profiling identified eight co-enriched KEGG pathways, among which arginine/proline metabolism, phagosome, oxidative phosphorylation and focal adhesion are consistently upregulated in resistant individuals. These findings suggest that effective resistance may involve a coordinated, multi-layered defense program-a proposed strategy that may minimizes immunopathology while facilitating pathogen clearance. From a practical perspective, the cross-omics validated molecules (e.g., *P4ha1*, COX6B, RAB5A, *CAV3*) serve as promising candidate biomarkers for DNA-based marker-assisted selection. Moreover, the pronounced enrichment of arginine/proline metabolism provides a testable hypothesis that should be evaluated in controlled feeding trials before any dietary recommendations are made. Our work deepens the understanding of host–pathogen interactions in marine teleosts and offers a foundation for potential application in breeding programs for VWND-resistant *L. crocea*.

## Figures and Tables

**Table 1:** Common KEGG pathways co-enriched in the transcriptome and proteome of the liver between the RL and SL groups.

Pathway	Function Categories	Transcriptome	Proteomics	AdjustPValue	Function Prediction
Arginine and proline metabolism	Amino acid metabolism	*P4ha1* (+) *Hoga1* (+)	Arginase (+) Creatine kinase (+)	0.042	Promotes NO synthesis for antibacterial activity, accelerates liver tissue repair, and attenuates pathogen-induced injury.
Cytokine–cytokine receptor interaction	Immune signaling	*Epo* (+) *Bmpr1b* (+)	CCL19 (+)	0.017	Maintains pro-/anti-inflammatory homeostasis and prevents excessive inflammatory storm.
Apoptosis	Cell death	*COX* (+)	Calpain-2 (+)	0.023	Mediates moderate apoptosis to eliminate infected liver cells without widespread tissue damage.
mTOR signaling pathway	Signal transduction	*Wnt7b* (−)	LPIN (+) V-ATPase (+) SEC13 (+)	0.035	Coordinates energy metabolism and autophagy to sustain cellular homeostasis during infection.
Oxidative phosphorylation	Energy metabolism	*COX6B* (+)	COX6B (+) QCR6 (+) V-ATPase (+)	0.008	Enhances mitochondrial ATP production to support energy supply for immune defense.
Glycolysis/Gluconeogenesis	Carbohydrate metabolism	*ldh* (+) *G6pc* (−)	ALDO (+)	0.026	Accelerates glucose mobilization and preferentially fuels immune-related processes.
Phagosome	Immune clearance	*TUB2* (−)	RAB5A (+) V-ATPase (+)	0.033	Strengthens phagocytic activity to clear intracellular pathogens and reduce liver bacterial load.
Focal adhesion	cell adhesion	*CAV3* (+)	CAPN2 (+) ARHGAP35 (+) SHC2 (+)	0.011	Preserves liver structural integrity and stabilizes cell–cell junctions.

## Data Availability

The data that supports the findings of this study are included in this published article and its supplementary information.

## Supplementary Materials

The following supporting information can be downloaded at https://www.mdpi.com/article/10.3390/ijms27167208/s1.
