# Supplementary material for: Integrated Liver Transcriptomic and Proteomic Analysis Reveals Resistance Mechanisms Against Pseudomonas plecoglossicida in Larimichthys crocea
Source: Int J Mol Sci. 2026 Aug 12;27(16):7208. doi: 10.3390/ijms27167208 (PMC13513298; doi:10.3390/ijms27167208)
Supplement: Supplementary file 1 [file ijms-27-07208-s001.zip › Supplementary Table S1.pdf]

Supplementary Table S1

Resequencing results statistics.

Sample	Raw Reads	Raw Q30 Rate (%)	Clean Reads	Total Mapped	Mapped to Gene
RL1	51671338	94.46	48357200	44163837 (91.33%)	26102681 (69.57%)
RL2	48404230	94.51	45395456	40985168 (90.28%)	22029462 (64.99%)
RL3	46153330	93.97	43258202	39352989 (90.97%)	21268115 (62.14%)
SL1	43045550	93.68	40343138	36299222 (89.98%)	21691271 (68.38%)
SL2	43732878	94.08	40936456	36784855 (89.86%)	21109573 (67.05%)
SL3	46680350	93.21	43704546	38605476 (88.33%)	22922666 (67.93%)
