# Supplementary material for: Integrated Liver Transcriptomic and Proteomic Analysis Reveals Resistance Mechanisms Against Pseudomonas plecoglossicida in Larimichthys crocea
Source: Int J Mol Sci. 2026 Aug 12;27(16):7208. doi: 10.3390/ijms27167208 (PMC13513298; doi:10.3390/ijms27167208)
Supplement: Supplementary file 1 [file ijms-27-07208-s001.zip › Supplementary Table S2.pdf]

Supplementary Table S2

Primers used in this study.

Gene	Nucleotide sequence (5'-3')	
COC	Forward	TCTATTGCGTTCTGTTG
	Reverse	AAGTAGGGTCTGGTAGTTAT
PAI1	Forward	TCAGTGGGTCCTTACAGC
	Reverse	CCAGCACCAGTTTCTCC
K1kb1	Forward	AACGGGAACTTCTGGTG
	Reverse	TTGGCTCTTATCTTTGGTC
P4ha1	Forward	ACCCGTCAGGCAGCAAG
	Reverse	TAGCCAGTGAGCCAAGC
TUB2	Forward	ACAACACCCACCTACGG
	Reverse	GGTCAGAGGAGCAAAGC
ldha	Forward	GACTCGGCACGCTTTCG
	Reverse	GTGGGTGTCCTTCCAGTTCTC
EPO	Forward	CGCAGAAGTCGCTATGA
	Reverse	AGTTGAGGGCCTGTTGTA
Repo2	Forward	TGGAGTCCGAACAACAG
	Reverse	CGAGCAGCCTTTACAGAT
CAV3	Forward	AGGTGGAGTTTGAGGATG
	Reverse	GGGATACCGAAGACAGC
BMPR1B	Forward	GTTGCCATAATCGTCTTCT
	Reverse	GCTTTGCGACTGCTCTA
β-actin	Forward	CCTTCACCACCACAGCCGAG
	Reverse	ATTCCGCAAGATTCCATACCGA
